# Preparation of High-Performance CdS@C Catalyst Using Cd-Enriched Biochar Recycled From Plating Wastewater

**DOI:** 10.3389/fchem.2020.00140

**Published:** 2020-03-17

**Authors:** Rui-Zhi Xing, Jia-Xin Li, Xing-Gui Yang, Ze-Wei Chen, Rong Huang, Zhi-Xuan Chen, Shun-Gui Zhou, Zhi Chen

**Affiliations:** Fujian Provincial Key Laboratory of Soil Environmental Health and Regulation, College of Resources and Environment, Fujian Agriculture and Forestry University, Fuzhou, China

**Keywords:** biochar, photocatalyst, adsorption, cadmium sulfide, hazardous waste

## Abstract

Biochar is widely used for the adsorptive removal of Cd from water and soil, but the Cd-enriched biochar produced carries a risk of secondary pollution. In this work, biochar derived from rice straw was used to adsorb Cd from plating wastewater. The Cd-enriched biochar showed a saturated adsorption capacity of about 63.5 mg/g and could be recycled and used in a mesoporous carbon-supported CdS (CdS@C) photocatalyst after pyrolysis carbonization and a hydrothermal reaction. The results demonstrated that the as-prepared CdS@C photocatalyst contained mixed cubic and hexagonal CdS phases, with a considerably lower band gap (2.1 eV) than pure CdS (2.6 eV). CdS@C exhibited an enhanced photocatalytic performance for the degradation of organic dyes under visible light irradiation compared with pure CdS due to its excellent light-harvesting capacity and efficient electron-hole separation. Moreover, the continuous formation of active species (h^+^, •OH, and O_2_•^−^) was responsible for the photodegradation of organic dyes using CdS@C. This work provides new insights for the safe disposal of Cd-enriched wastewater and for improving the economic viability of Cd-contaminated resources by recovering a value-added photocatalyst.

## Introduction

Cadmium (Cd) is extremely toxic to plants and poses a serious threat to humans and animals when it enters the food chain (Clemens et al., 2013; Zhao et al., 2015; Gong et al., 2019; Hussain et al., 2019). It is introduced into the environment through the discharge of Cd-containing wastewater from industrial processes involved in electroplating, photovoltaics, and metal smelting (Hayat et al., 2019; Nain and Kumar, 2019; Król et al., 2020). Biochar is an ideal adsorbent for the removal of heavy metals from aqueous environments due to its low cost, widespread availability, eco-friendliness, and high adsorption capacity (Inyang et al., 2012; Harsono et al., 2013; Xu et al., 2013; Chen et al., 2018; Ahmed et al., 2019). In fact, biochar derived from different biomass materials has been investigated to develop alternative adsorption techniques for the removal of Cd from contaminated environments (Chen et al., 2015, 2018, 2019a; Wu et al., 2019). The majority of studies have focused on the modification of biochar to enhance its adsorption capacity (Chen et al., 2015, 2018, 2019b; Wu et al., 2019). However, fewer studies have attempted to identify an appropriate strategy for the disposal and upcycling of Cd-enriched biochar after adsorption, which poses a secondary pollution risk. The Cd accumulated on biochar can be released back into surrounding environments by leaching or other natural processes when it is disposed of in a landfill (He et al., 2019; Wang et al., 2019; Zhang et al., 2020). Thus, there is a need to identify a safe and economically viable disposal method for Cd-enriched biochar.

A promising disposal method for metal-enriched hazardous waste is upcycling in the form of high value-added products (Harumain et al., 2017; Cui et al., 2018; Ye et al., 2019). In principle, noble metals (e.g., Ni, Au, Cu, and Pt) can be directly recovered by incineration/pyrolysis or leaching (Keller et al., 2005; Zhang et al., 2014; Guilpain et al., 2018). However, Cd is not a valuable heavy metal, and it is not economically viable to extract it directly from Cd-accumulated hazardous waste (Sadegh et al., 2007; Chen et al., 2019c). Interestingly, some metal-enriched hazardous wastes have shown great potential for upcycling as catalysts (Parker et al., 2014; Harumain et al., 2017; Chen et al., 2019c,d). For example, palladium in metal-enriched hazardous wastes can be recovered in the form of high-value carbon-supported catalysts (Parker et al., 2014; Harumain et al., 2017). In addition, our previous works have suggested that Cd-enriched biomass can be transformed into a CdS@C composite with high photocatalytic performance (Chen et al., 2019c,d). The carbon carrier prevented the aggregation and photocorrosion of catalyst particles and facilitated the transport and contact of substrates between the support and the active sites (Gao et al., 2011; Han et al., 2011; Huang et al., 2018; Chen et al., 2019c). In light of these advantages, biochar-supported CdS composites may greatly enhance the photocatalytic activity and stability of the catalysts (Ma et al., 2017; Huang et al., 2018, 2019; Bantawal et al., 2019; Cao et al., 2019; Hu et al., 2019).

Biochar has been widely used for Cd adsorption or immobilization in environmental remediation because it is capable of adsorbing high levels of Cd (>1%, dry weight) (Chen et al., 2015, 2018, 2019b; Wu et al., 2019). However, no research has been conducted to identify a feasible way to recycle Cd-enriched biochar into a catalyst. Such a method could achieve the dual effect of high-value utilization and secondary pollution risk management. In this work, we developed a facile and low-cost method for the preparation of a CdS@C photocatalyst by recycling Cd-enriched biochar after treatment of Cd-containing plating wastewater. The photocatalytic performance and photodegradation mechanism of CdS@C for the degradation of organic pollutants were investigated.

## Experimental

### Materials

Sodium sulfide (Na_2_S·2.5H_2_O, ≥98%), rhodamine B (RhB, 95%), methylene blue (MB, 95%), acid red 11 (AR11, 96%), and 5,5′–dimethyl–1–pyrroline–N–oxide (DMPO, 97%) were commercially purchased. Deionized water was used to prepare reaction and stock solutions. The rice straw biomass was picked from the College of Life Sciences, Fujian Agriculture and Forestry University (Figure S1). The cadmium plating wastewater was taken from an electroplating factory in Quanzhou, Fujian Province, China. The physiochemical parameters of the cadmium plating wastewater are listed in Table S1.

### Fabrication of CdS@C Composite Photocatalyst

The rice straw biomass was washed with deionized water and dried for 12 h in an oven at 105°C. The rice straw was then ground into a powder of about 100 mesh and carbonized in a tube furnace at 700°C under a nitrogen atmosphere for 3 h to obtain biochar capable of adsorbing Cd. About 0.5 g biochar was added into 150 mL Cd-containing plating wastewater and stirred with a magnetic stirrer for 40 min. After adsorption, Cd-enriched biochar (denoted as Cd-Biochar) was obtained through centrifugation, followed by washing with deionized water and drying at 60°C for 24 h.

Under an N_2_/O_2_ (6/1, v/v) mixed atmosphere, Cd-Biochar was oxidized at 650°C for 0.5 h, which increased the cadmium content in the biochar from 2.5 to 5%. This was dispersed into a 5 mM Na_2_S solution in a Teflon-lined stainless steel pot, heated in a muffle furnace at 180°C for 72 h, and then cooled to ambient temperature. The CdS@C composite photocatalyst was obtained after washing with deionized water and filtering (Chen et al., 2019c).

### Characterization

Scanning electron microscopy (SEM) images were acquired using a JSM6700-F (JEOL, Japan) operating at 10 kV to compare the surface morphology of biochar before and after synthesis. Inductively coupled plasma mass spectrometry (ICP-MS) and elemental analysis (EA) were used to determine the contents of C, H, S, and Cd in the material. Transmission electron microscopy (TEM) and high-resolution TEM (HRTEM) images were used to determine the crystal morphology and phase of the materials using a JEM-2010 at 200 kV. X-ray powder diffraction (XRD) patterns were used to determine the composition and molecular structure of the materials; these patterns were obtained using the Ultima IV (Rigaku, Japan), operating at a 40-kV tube voltage and a 40-mA tube current. X-ray photoelectron spectroscopy (XPS) was carried out to analyze the valence states of elements using an ESCALAB 250 (Thermo Fisher, America) with an Al/Mg double anode target as the radiation source (15 kV, 10 mA). The C 1s peak at 284.7 eV was used to calibrate the energy scale of the XPS spectra. The diffuse reflectance spectra (DRS) were recorded from 200 to 800 nm on an UV2550 UV-Vis spectromater (Shimadzu, Japan) using a BaSO_4_ background. The band gap energy (*E*_*g*_) of the photocatalyst was calculated by the Kubelka-Munk (K-M) formula:

$$\begin{array}{l} {\left(Ahv/K\right)}^{2}=h_{v}-E_{g}. \end{array}$$

where *A* is the absorbance, *h*_v_ is 1,240/wavelength, and *K* is a constant. The electron spin resonance (ESR) spectra were obtained of active substances produced by visible light irradiation using a Bruker A300 spectrometer (Bruker, Germany).

### Photocatalytic Degradation of Organic Dyes

The role of RhB in photocatalytic degradation was studied. In a general catalysis procedure, photocatalyst (20.0 mg) was dispersed in 100 mL of a photocatalytic reactor with a 50 mL RhB solution (with a final RhB concentration of 40.0 mg/L). The samples were magnetically stirred at 800 rpm for 30 min in the dark. A xenon lamp (CEL-HXF300, Beijing) equipped with cut-off filters at 420 and 780 nm was used as the light source. After visible light irradiation for a certain period of time, the photocatalytic absorbance experiments were monitored by a 554-nm ultraviolet-visible spectrophotometer. The efficiency of photocatalysis was calculated by the following formula:

$$\begin{array}{l} R=C/C_{\text{0}}\times \;100\% \end{array}$$

where *R* is the degradation rate, *C*_*0*_ is the characteristic absorbance of RhB before photocatalysis, and *C* is the characteristic absorbance of RhB after photocatalysis (Huang et al., 2017).

### Photocatalytic Mechanism Analysis

The current vs. time (I-t) curves were obtained using a Shanghai Chenhua CHI600E electrochemical workstation. First, 5 mg of a sample was ultrasonically dispersed in a 5% naphthene solution. About 5 μl of the suspension was then uniformly smeared on an ITO glass substrate (1 cm × 1 cm) and allowed to dry at room temperature. The coating process was repeated 10 times to form a thin film on the ITO glass substrate. The change in the instantaneous current with time was used to evaluate whether the photocatalyst had a photoelectric effect. An electron spin resonance (ESR) spectrometer (Bruker A300) was used to detect the presence of superoxide radical (O_2_•^−^) during photocatalytic reactions using the capture agent DMPO. Hydroxyl radicals (•OH) generated during the photocatalytic reaction could react with terephthalic acid (TA) to produce highly fluorescent 2-hydroxyterephthalic acid (TAOH), and the fluorescence was detected by fluorescence spectroscopy.

## Results and Discussion

### Preparation and Characterization of CdS@C Photocatalyst

**Scheme 1:**
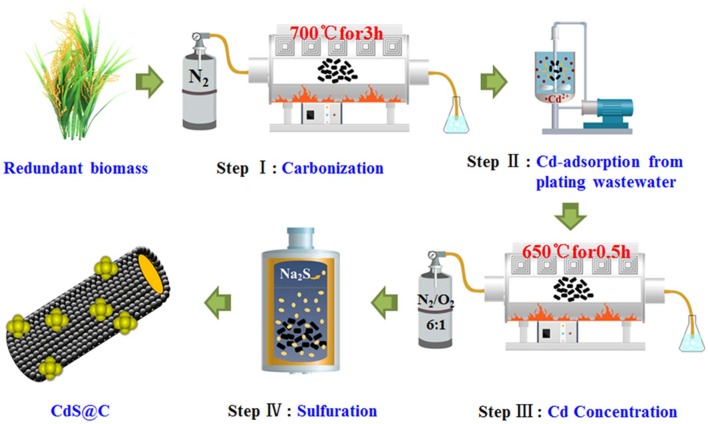
Diagram of the route for CdS@C synthesis route.

Rice straw biochar was used to recycle Cd from plating wastewater and then converted into a CdS@C composite using the route shown in Scheme 1. The Cd equilibrium adsorption of the biochar was achieved in 30–40 min and reached about 63.5 mg/g (Figure S2). The Cd-Biochar was further converted into a CdS@C composite in a stepwise process, as reported in our previous works (Chen et al., 2019c,d). The secondary pyrolysis of Cd-Biochar was conducted under an N_2_/O_2_ (6/1, v/v) atmosphere at 650°C before the sulfuration (Chen et al., 2019c). The elemental analysis indicated that the S and Cd contents were ca. 4.4% and ca. 16.1%, respectively, in the synthetic material after Cd concentration and sulfuration (Table 1).

**Table 1:** Elemental analysis of the Cd-Biochar and CdS@C.

**Samples**	**Element**			
	**C (%)**	**H (%)**	**S (%)**	**Cd (%)**
Cd-Biochar	55.7	1.1	0.4	6.3
CdS@C	21.4	1.0	4.4	16.1

**Figure 1:**
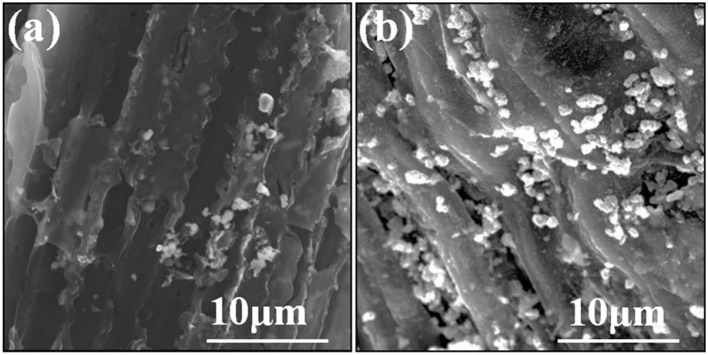
SEM images of Cd-Biochar **(a)** and CdS@C **(b)**.

The microscopic surface texture of Cd-Biochar and as-prepared CdS@C composite were investigated by SEM. The Cd-Biochar exhibited obvious porous tubular structures with a rough and hierarchical surface (Figure 1a). After Cd concentration and sulfuration, many uniformly distributed particles were observed on the surface of the as-prepared CdS@C composite (Figure 1b). The EDX elemental analysis suggested that the particles on the surface of the composite were mainly composed of S and Cd (Figure S3).

**Figure 2:**
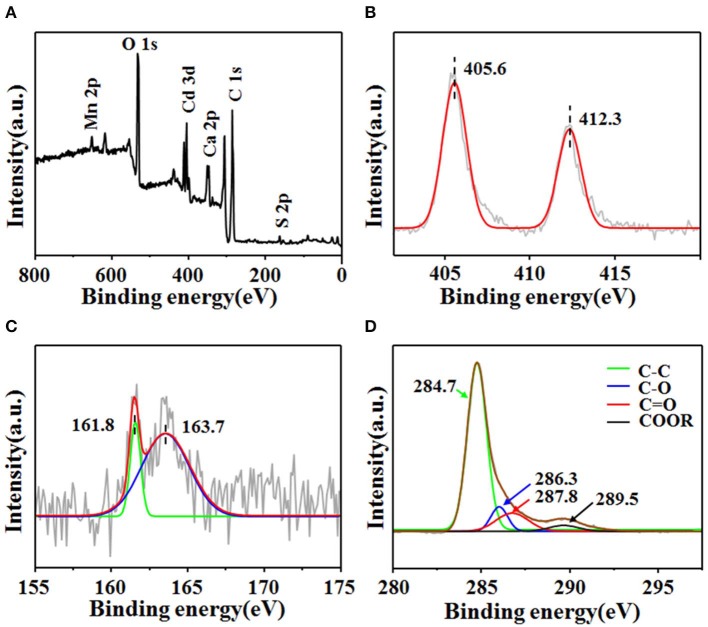
XPS spectral of CdS@C **(A)** and high-resolution XPS spectra of Cd 3d **(B)**, S 2p **(C)**, and C 1s **(D)**.

The chemical composition, the valence state of the elements, and surface functionality groups of CdS@C were further characterized by XPS. As shown in Figure 2, the wide-scan XPS spectra of the composite contain Cd 3d, S 2p, O 1s, and C 1s signals (Figure 2A). The deconvoluted high-resolution spectrum demonstrated that the Cd 3d spectrum contained peaks at 405.6 eV (Cd 3d_5/2_) and 412.3 eV (Cd 3d_3/2_), which was consistent with the characteristic peaks of Cd^2+^ in CdS (Figure 2B) (Huang et al., 2018; Chen et al., 2019c). The deconvoluted S 2p_3/2_ and S 2p_1/2_ peaks at 161.8 and 163.7 eV indicated the presence of S^2−^ in the composites (Figure 2C) (Ye et al., 2012; Huang et al., 2018). The Cd 3d and S 2p XPS spectra confirmed the presence of CdS in the as-prepared composite. The C1s peak-fitting showed four peaks at 284.7, 286.3, 287.8, and 289.5 eV, respectively (Figure 2D). The peak at 284.7 eV was assigned to the sp^2^ carbon species, while the peaks at 286.3, 287.8, and 289.5 eV were identified as oxygenated carbon species (i.e., C–O, C=O, and COOR) (Okpalugo et al., 2005; Ye et al., 2012). The binding energies in the high-resolution C1s XPS spectrum suggested that the carbon support was functionalized with oxygen-containing groups. Additionally, the wide-scan XPS spectrum also confirmed the presence of other mineral elements, such as Mn and Ca (Figure 2D).

**Figure 3:**
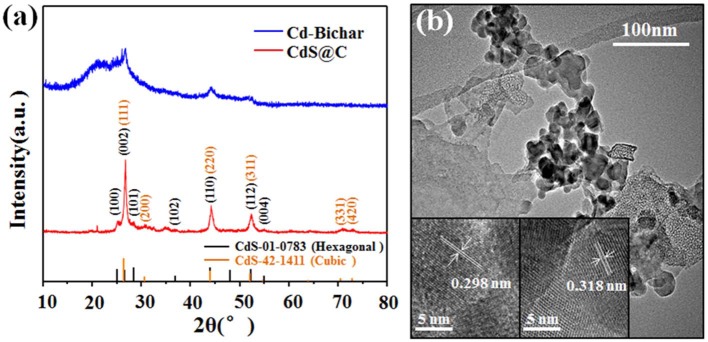
XRD patterns of the Cd-Biochar and CdS@C composite materials **(a)**; TEM image of CdS@C **(b)** (insets are HRTEM images).

XRD and TEM were used to confirm the presence of CdS in the composite. As shown in Figure 3a, the Cd-Biochar and the composite showed different characteristic peaks in their XRD patterns. The diffraction peaks of the composite exhibited a mixture of cubic (PDF 42-1411) and hexagonal phase (PDF 01-0783) CdS. In addition, the HRTEM images of the CdS particles on the composite showed two different crystal lattice spacings of 0.298 and 0.318 nm, which were assigned to the characteristic (111) and (101) crystal planes of cubic and hexagonal CdS, respectively (Figure 3b). Thus, XPS, XRD, and TEM results verified that the Cd-Biochar was recycled in the form of CdS@C composite. It is worth noting that the CdS@C composite was prepared by recycling of Cd from plating wastewater. Additionally, Cd-enriched hyperaccumulator biomass could also be used to synthesize a CdS@C photocatalyst, as reported in our previous works (Chen et al., 2019c,d). This study suggests that CdS@C can be synthesized using a facile and low-cost method by upcycling different types of Cd-enriched biomasses.

**Figure 4:**
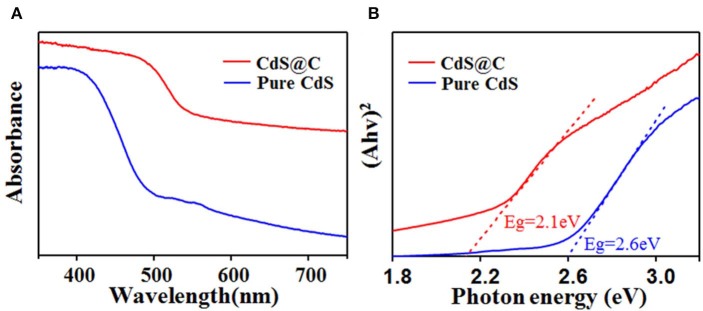
DRS spectra **(A)** and K-M plots **(B)** of pure CdS and CdS@C.

The visible-light absorption properties of CdS@C composite were investigated by UV-Vis diffuse reflectance spectroscopy (DRS) to evaluate its photocatalytic activity. As shown in Figure 4A, compared with pure CdS, the light absorption of CdS@C from 350 to 750 nm was increased, suggesting a more efficient light-harvesting of CdS@C in photocatalysis under visible irradiation (Apte et al., 2012). According to the Kubelka-Munk formula, the bandgap energy of the CdS@C was 2.1 eV, which was lower than that of pure CdS (ca. 2.6 eV) (Figure 4B), suggesting a better photocatalytic performance under visible light irradiation. The bandgap energy of CdS@C was similar to a previously reported CdS composite catalyst prepared by loading hydrothermally prepared CdS precursors on biochar (Zhu et al., 2017).

### Photocatalytic Properties of CdS@C in Organic Dyes Degradation

**Figure 5:**
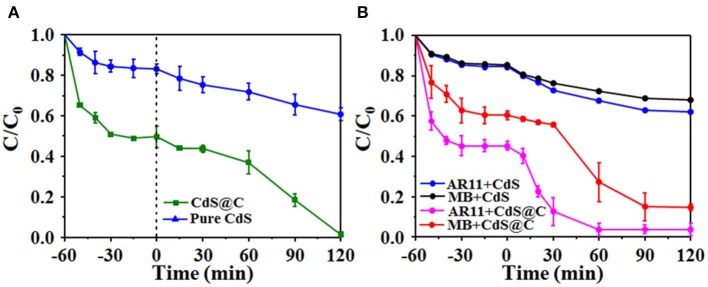
Degradation efficiency of photocatalytic RhB dyes under visible light **(A)**; MB and AR11 degradation efficiency of CdS and CdS@C **(B)**.

Previous works have demonstrated that mixed-phase CdS composites supported on porous materials have strong photocatalytic activities (Dai et al., 2014; Li et al., 2016). The photocatalytic activity of CdS@C was evaluated by the photodegradation of organic dyes under visible light irradiation. As shown in Figure 5, the photocatalytic efficiency of CdS@C was generally significantly higher than that of pure CdS. The RhB photodegradation of CdS@C was about 98.6% after irradiation for 120 min, while the RhB photodegradation efficiency using pure CdS was considerably lower at about 39.1% under the same conditions (Figure 5A). The photodegradation of MB and AR11 confirmed that the photocatalytic activity of CdS@C was enhanced compared with pure CdS (Figure 5B). Additionally, the photodegradation efficiency of RhB and MB was considerably higher than that of AR11 under photocatalysis using CdS@C. This may be due to the fact that RhB and MB are cationic dyes, whereas AR11 is anionic. Electrostatic adsorption between cationic dyes (i.e., RhB and MB) and the negatively charged CdS@C could promote favorable transport and contact between organic dyes during photocatalysis (Lv et al., 2005; Mahmoodi et al., 2011; Mittal and Khanuja, 2019). It was believed that the enhanced photocatalytic activity of CdS@C was attributable to improved substrate accessibility, light-harvesting efficiency, and electron-hole separation due to the porous carbonaceous carrier (Huang et al., 2018).

**Figure 6:**
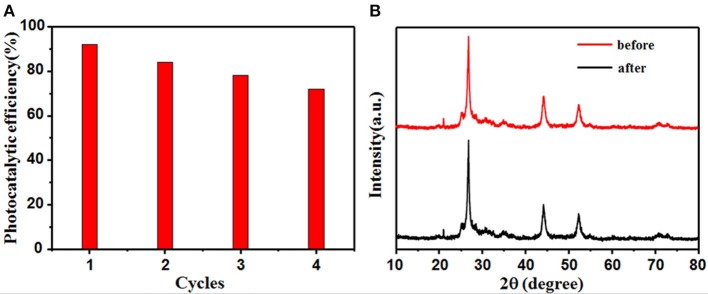
CdS@C stability after 4 reactions of RhB photodegradation **(A)**; XRD pattern of CdS@C before and after the recycling reactions **(B)**.

For practical applications, an ideal photocatalyst will have high durability in addition to high photoactivity. The reusability and stability of CdS@C were further tested by recycling it after RhB degradation. Figure 6A shows that the photocatalytic activity remained above 70% of its initial value after four consecutive degradation cycles. The decreased photocatalytic activity may have been due to the deactivation and agglomeration of CdS, as well as the loss of CdS@C photocatalyst during the water washing-drying process. No significant changes were detected in the XRD patterns of CdS@C before and after 4 cycles of RhB photodegradation (Figure 6B). This indicates that the structure and composition of CdS@C were stable during photocatalysis, suggesting that the composite had good reusability and stability.

### Photocatalytic Degradation Mechanism of Organic Dyes Using CdS@C

**Figure 7:**
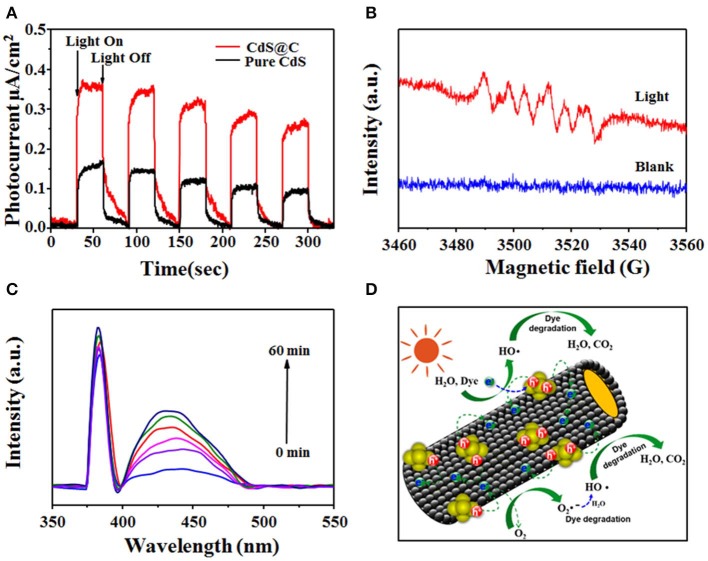
Transient photocurrent spectra of pure CdS and CdS@C under visible light **(A)**; ESR spectra of the superoxide radical captured by DMPO for CdS@C **(B)**; the time-dependent fluorescence intensity of TAOH using CdS@C under visible irradiation **(C)**; photocatalytic degradation mechanism of CdS@C **(D)**.

It was speculated that the photogenerated electron-hole pairs of CdS could be efficiently separated by the biochar carrier, leading to enhanced photocatalytic performance of the CdS@C composite (Lei et al., 2017). The transient photocurrent responses were measured to assess the generation and transport of photogenerated electrons during photocatalysis using CdS@C under intermittent visible-light illumination. As shown in Figure 7A, a photocurrent was rapidly generated and remained stable for 30 s during illumination, and immediately decreased to zero when illumination was stopped. Obviously, the photocurrent of the CdS@C was significantly higher than that of pure CdS under the same irradiation conditions, indicating an enhanced separation and transport of electron-hole pairs (Li et al., 2018). As a consequence, the charge recombination was reduced, and more reactive species such as *h*^+^ and O_2_•^−^, were produced. Additionally, *h*^+^ and O_2_•^−^ could produce •OH. To verify the presence of •OH and O_2_•^−^ during photocatalysis using CdS@C, ESR and time-dependent fluorescence spectra were obtained by combining the composite with radical scavengers (i.e., DMPO, TA). As shown in Figure 7B, the characteristic peaks of the DMPO–O_2_•^−^ were detected after light irradiation of CdS@C, whereas the ESR signal of DMPO–O_2_•^−^ remained silent under dark conditions (Li et al., 2019a, 2020). Moreover, the time-dependent fluorescence spectra of TAOH also confirmed the presence of •OH during photocatalysis of the CdS@C, and the fluorescence intensity increased proportionally with the production of •OH (Figure 7C) (Li et al., 2019b).

Based on the above research, a possible photodegradation mechanism of the dyes was proposed. As illustrated in Figure 7D, due to the low band gap of CdS@C, visible light was efficiently harvested and used to generate electron-hole pairs. Electron-hole pairs were efficiently separated without recombination due to the good conductivity of the mesoporous biochar support and then participated in the formation of various active species, which participated in the photodegradation of dyes (Zhang et al., 2017; Huang et al., 2018; Li et al., 2019b, 2020).

## Conclusion

This work presents the first report of the synthesis of a CdS@C composite photocatalyst by upcycling Cd from plating wastewater. The photocatalytic performance of the composite catalyst obtained was evaluated by using it in the photodegradation of organic dyes. CdS@C contained a mixture of cubic and hexagonal CdS and exhibited a considerably higher light-harvesting capacity and electron-hole separation efficiency. CdS@C displayed enhanced photocatalytic efficiency toward the degradation of organic dyes compared with pure CdS. Further investigation of the photocatalytic mechanism suggested that the photodegradation of organic dyes was largely attributable to the continuous formation of catalytically active species (*h*^+^, •OH, and O_2_•^−^) during the photocatalysis of CdS@C.

## Data Availability Statement

The datasets generated for this study are available on request to the corresponding author.

## Author Contributions

ZC, S-GZ, and R-ZX designed the research and co-wrote the paper. R-ZX, X-GY, Z-WC, and J-XL conducted experiments and characterized the materials. RH and Z-XC conducted the catalysis.

### Conflict of Interest

The authors declare that the research was conducted in the absence of any commercial or financial relationships that could be construed as a potential conflict of interest.
