# Supplementary material for: Preparation of High-Performance CdS@C Catalyst Using Cd-Enriched Biochar Recycled From Plating Wastewater
Source: Front Chem. 2020 Mar 17;8:140. doi: 10.3389/fchem.2020.00140 (PMC7089938; doi:10.3389/fchem.2020.00140)
Supplement: Supplementary file 1 [file Data_Sheet_1.PDF]

Supporting Information

Preparation of high performance CdS@C catalyst by recycling of Cd from plating wastewater

Rui-Zhi Xing[1], Jia-Xin Li[1], Xing-Gui Yang[1], Ze-Wei Chen[1], Rong Huang[1], Zhi-Xuan Chen[1], Shun-Gui Zhou[1], Zhi Chen[1*]

Fujian Provincial Key Laboratory of Soil Environmental Health and Regulation, College of Resources and Environment, Fujian Agriculture and Forestry University, No. 15 Shang Xia Dian Road, Fuzhou, Fujian350002, China

* Corresponding author: Dr Zhi Chen

Email: chenzhi0529@163.com, Phone: +86-590-86398509

Number of pages: 4

Number of figures: 3

Figure list

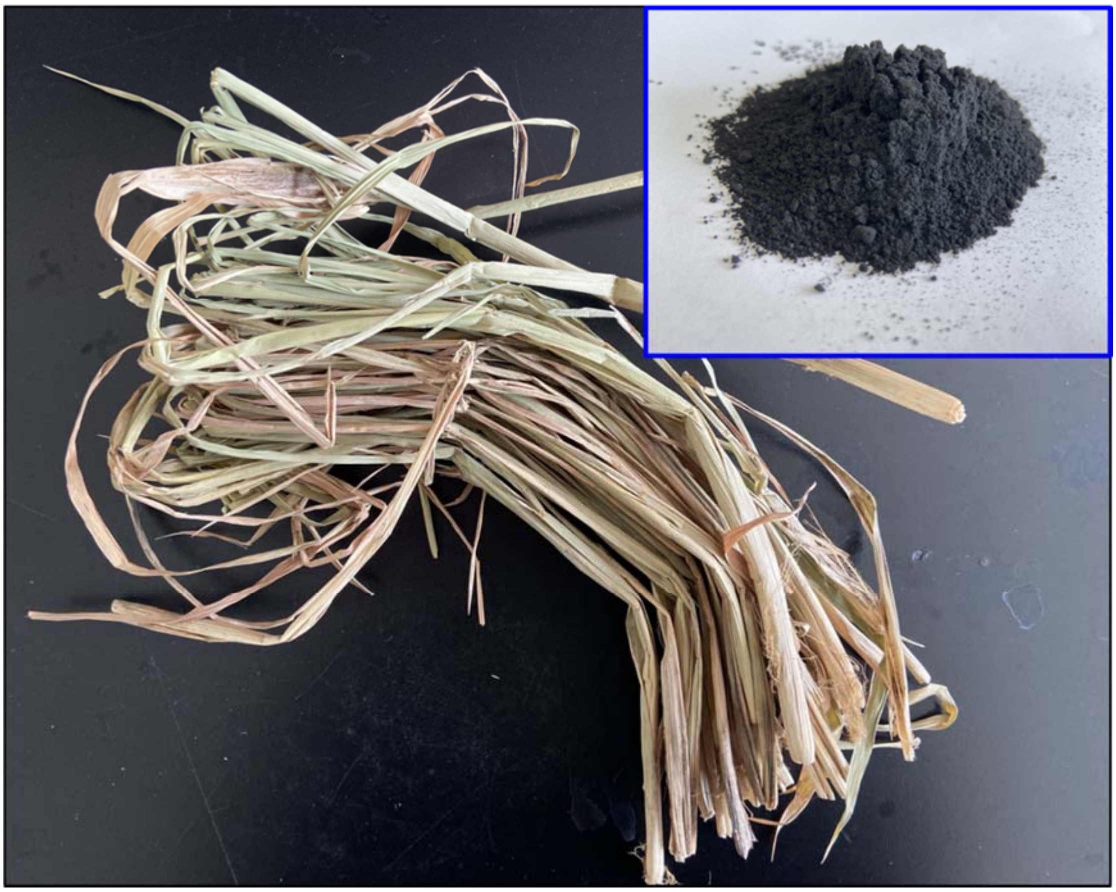

FIGURE S1 The biomass of rice straw (inset: biochar prepared from rice straw).

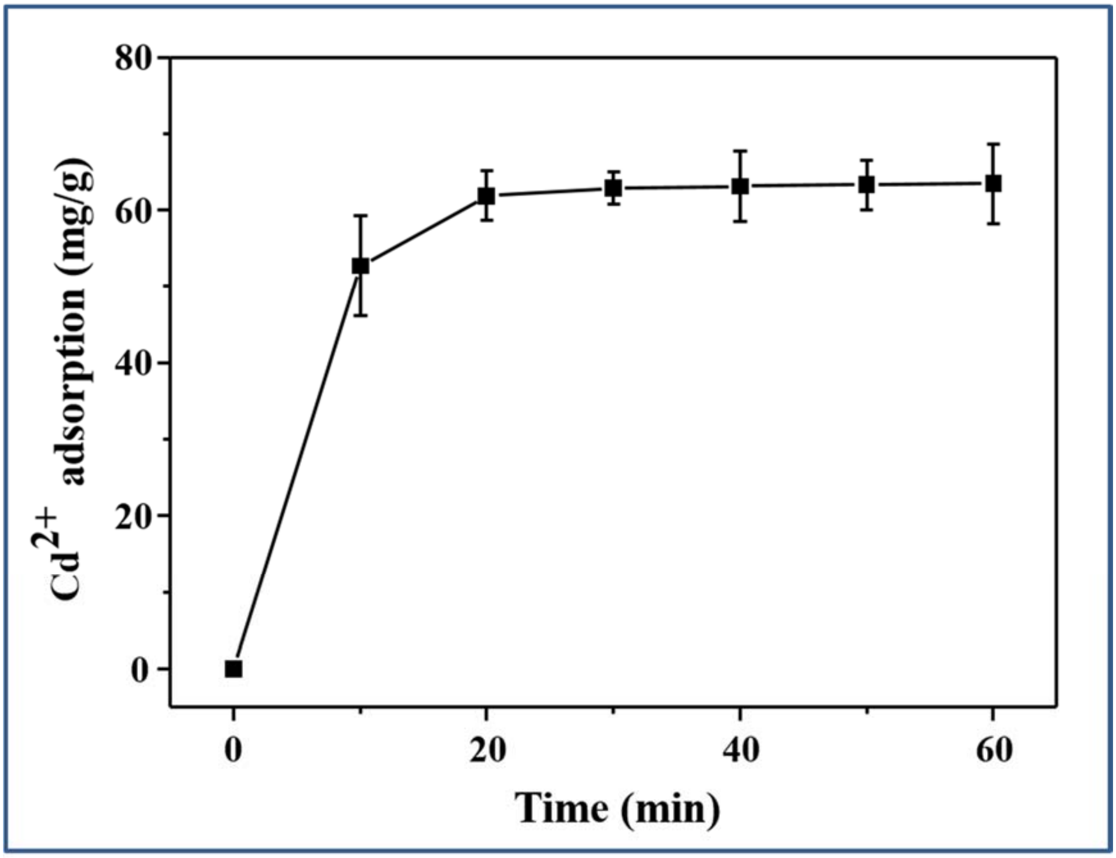

FIGURE S2 Adsorption curve of biochar derive from rice straw

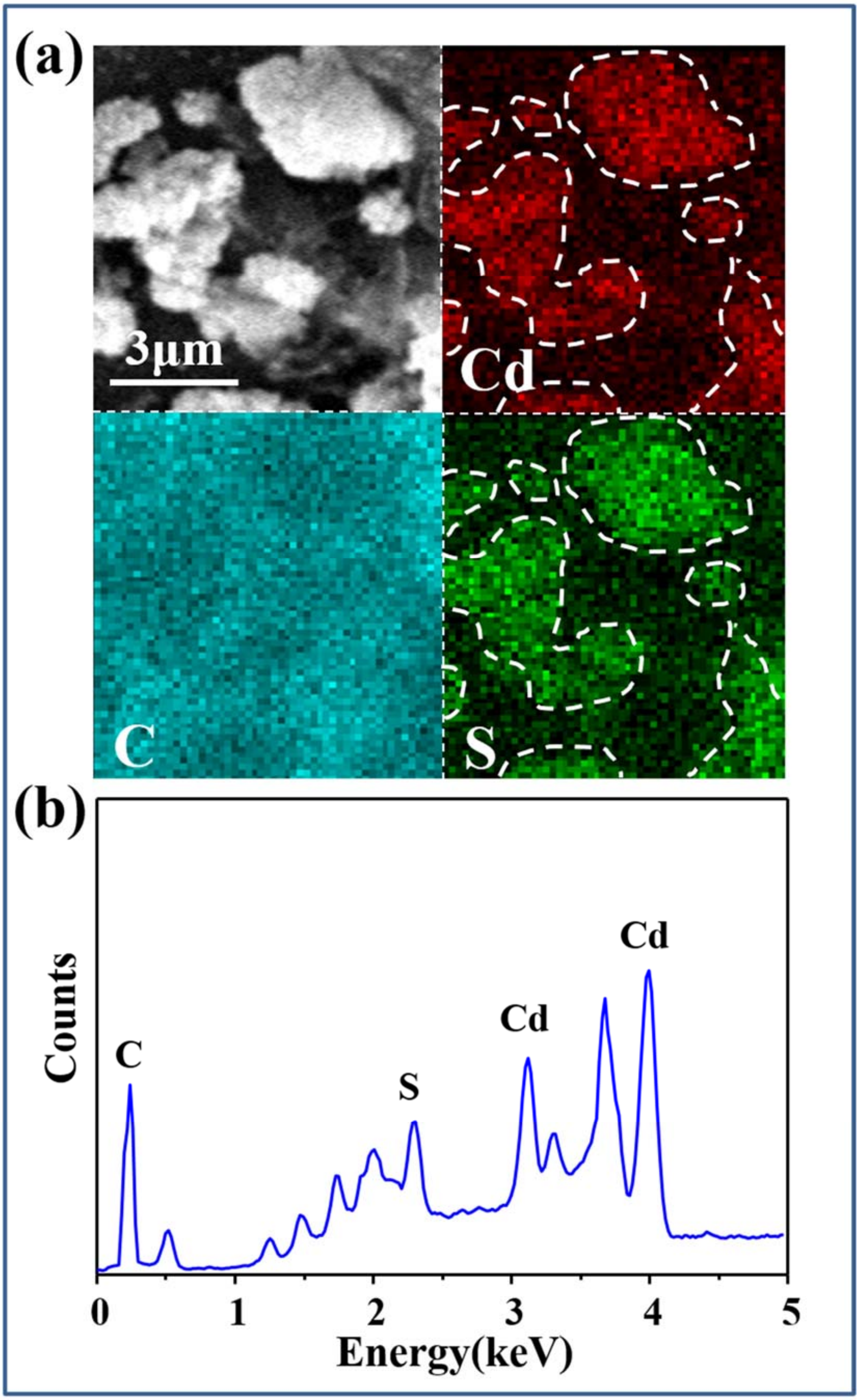

FIGURE S3 (a) Surface scanning of the CdS@C and (b) EDX elemental analysis
